# A practical approach for continuous in situ characterization of radiation quality factors in space

**DOI:** 10.1038/s41598-022-04937-1

**Published:** 2022-01-27

**Authors:** Igor Shuryak, Tony C. Slaba, Ianik Plante, Floriane Poignant, Steven R. Blattnig, David J. Brenner

**Affiliations:** 1 Center for Radiological Research, Columbia University Irving Medical Center, 630 West 168th St., New York, NY 10032 USA; 2 NASA Langley Research Center, Hampton, VA 23681 USA; 3 KBR, Houston, TX 77058 USA; 4 National Institute of Aerospace, Hampton, VA 23666 USA

**Keywords:** Computational biophysics, Space physics, Risk factors

## Abstract

The space radiation environment is qualitatively different from Earth, and its radiation hazard is generally quantified relative to photons using quality factors that allow assessment of biologically-effective dose. Two approaches exist for estimating radiation quality factors in complex low/intermediate-dose radiation environments: one is a fluence-based risk cross-section approach, which requires very detailed in silico characterization of the radiation field and biological cross sections, and thus cannot realistically be used for in situ monitoring. By contrast, the microdosimetric approach, using measured (or calculated) distributions of microdosimetric energy deposition together with empirical biological weighting functions, is conceptually and practically simpler. To demonstrate feasibility of the microdosimetric approach, we estimated a biological weighting function for one specific endpoint, heavy-ion-induced tumorigenesis in APC^1638N/+^ mice, which was unfolded from experimental results after a variety of heavy ion exposures together with corresponding calculated heavy ion microdosimetric energy deposition spectra. Separate biological weighting functions were unfolded for targeted and non-targeted effects, and these differed substantially. We folded these biological weighting functions with microdosimetric energy deposition spectra for different space radiation environments, and conclude that the microdosimetric approach is indeed practical and, in conjunction with in-situ measurements of microdosimetric spectra, can allow continuous readout of biologically-effective dose during space flight.

## Introduction

The radiation environment in space, and on the Moon and the distant planets, is qualitatively and quantitatively very different from that on the Earth’s surface^1–3^. It consists of a mixture of different radiation types and energies that include protons, neutrons and heavy ions. Astronauts are exposed to this complex low/intermediate-dose radiation environment (with total expected doses < 1 Gy), the components of which can differ dramatically in their relative biological effectiveness^2,4,5^. For example, densely-ionizing energetic heavy ions and neutrons can have much higher biological effectiveness per unit dose than sparsely-ionizing x or γ rays^6,7^.

In practice, these increased health risks from exposure to space radiation are quantified using radiation quality factors^8,9^ or radiation weighting factors^10,11^, which are effectively multiplicative scaling factors used to characterize the increased health risks of densely ionizing radiations relative to the better quantified x- or γ-ray risks. For example, at low radiation doses, the radiation quality factor, *Q*, represents a multiplicative factor to scale from the physical quantity radiation dose (*D*, in Gray) to the dose equivalent (*H*, in Sieverts)^8,9^:

1 $$H = D \times Q.$$

A recent report from the National Academies of Sciences^12^ endorsed the use of the dose equivalent, *H*, for defining space flight lifetime radiation exposure limits.

Currently, two potential approaches exist to estimate radiation quality factors, and thus dose equivalents, in a complex low/medium dose radiation environment such as in space. One is the fluence-based risk cross-section approach^13–15^, which is used in the current NASA cancer risk model^16^. This method requires an extremely detailed characterization of the radiation field, including energy spectra and physical and biological action cross sections for every important radiation type that contributes to the overall radiation field. By contrast, the microdosimetric approach is both conceptually and practically simpler. In short, this approach generates quality factors using measured or calculated microdosimetric spectra (distributions of energy depositions within cellular-sized volumes) that are weighted with an empirically determined biological weighting function. Because microdosimetric energy distributions can be easily and continuously measured in space environments^17–20^, and assuming that an appropriate biological weighting function is available (the subject of this paper), then radiation quality factors, and thus, corresponding dose equivalents, can be continuously assessed in situ in space environments. This microdosimetric approach, originally suggested by Zaider and Brenner^21^ and Bond et al*.*^22^, and endorsed in the International Commission on Radiation Units and Measurements (ICRU) Report 40^9^, has been used extensively in other radiation exposure contexts^22–33^.

Both the fluence-based cross section approach and the microdosimetric approach have been extensively reviewed in a National Council on Radiation Protection and Measurements (NCRP) Report^34^. Direct intercomparisons there of these approaches suggest that they produce quality factor predictions that are within ~ 30% of each other.

In the present paper we examine the utility and practicality of the microdosimetric approach for characterizing radiation quality factors in space environments, by generating relevant biological weighting functions, in this example for an intestinal cancer endpoint. This approach can be applied to other relevant radiation-induced cancer (and potentially non-cancer) endpoints, provided there are experimental data available to calibrate it, allowing ultimately for the generation of a consensus average quality factor.

### The microdosimetric approach

The microdosimetric approach can be used to calculate the mean quality factor based on the measured (or calculated) distribution of microdosimetric energy depositions. This energy deposition distribution is then weighted using a biological weighting function to produce a mean quality factor. The logic behind this approach is that differences in biological effectiveness between different radiations at the same dose can only be caused by differences in the patterns of energy deposition on the microscopic scale, such as within cellular and sub-cellular targets. For example, the spatially-dense pattern of energy depositions from heavy ions more frequently generates severe, difficult to repair biological damage (e.g., complex DNA double strand breaks) per unit dose than does the pattern of spatial energy depositions from photons^35–37^. Relative health risks from exposure to different radiation types are thus determined by the corresponding different initial physical energy deposition patterns at the microscopic level, and these in turn can be characterized by the microdosimetric distribution of energy depositions.

Based on these considerations, the microdosimetric approach for calculating the mean quality factor, $\overline{Q}$, can be written as follows^21^:

2 $$\overline{Q} = \int {Q(y) d(y) dy,}$$

where *y* is the stochastic quantity lineal energy, defined as the energy deposited in a defined microscopic site by a single radiation track, divided by the mean path length in that site, *d*(*y*) is the normalized dose distribution (probability density) from single-event lineal energy depositions (the measured or calculated microdosimetric energy deposition distribution)^38^, and *Q*(*y*) is an empirical consensus-determined biological weighting function^21^.

The normalized dose distribution *d*(*y*) from single-event lineal energy depositions is, of course, radiation specific. It is based on the measured (or calculated) probability distribution of lineal energies deposited by each radiation type *i*, which is denoted by *f*_*i*_(*y*)^21^. The normalized dose-weighted lineal energy distribution *d*_*i*_(*y*) is derived from this frequency-weighted distribution *f*_*i*_(*y*) as follows^38^:

3 $$d_{i} (y) = y f_{i} (y)/\int {y f_{i} (y) dy } .$$

Here *f*_*i*_(*y*) and *d*_*i*_(*y*) represent probability densities of lineal energy frequencies and dose contributions, respectively^38^, and thus the total integral of each of these distributions over *y* is, by definition, unity.

In a space environment, *d*(*y*) can be continuously measured using a compact tissue-equivalent proportional counter^17,18,20^ or a silicon microdosimeter^19,39^. Thus, given an empirical consensus biological weighting function, *Q*(*y*), the mean quality factor, $\overline{Q}$, can be continuously assessed using Eq. (2), and thus the dose equivalent can be continuously assessed using Eq. (1).

Clearly, the key to this microdosimetric approach is to estimate the empirical consensus biological weighting function, *Q*(*y*) (see Eq. 2). As with all quality factors and radiation weighting factors, this function will represent a consensus “averaged” over a variety of relevant biological endpoints and health effects^21^. The notation that we use here, taken from Zaider and Brenner^21^, is that the corresponding biological weighting function for a specific biological or health effect, *ε*, is denoted by the lower case function, *q*_*ε*_(*y*)^21^, and the consensus weighted average over a variety of “relevant” *q*_*ε*_(*y*) functions is denoted by the upper case notation *Q*(*y*)*.* In the present study we demonstrate the concept by estimating *q*_*ε*_(*y*) for the endpoint of radiation-induced induction of intestinal tumors in APC^1638N/+^ mice.

### Estimation of *q*_ε_(*y*), for a specific biological endpoint, ε

As described below, we analyzed the results of a series of experiments with a given biological endpoint, *ε*. We define the metric ${\mathcal{E}}_{i}$ to represent the relative yield at low doses (vs. γ rays) of the given endpoint, *ε*, induced by a given radiation type *i*, which is characterized by microdosimetric spectra *d*_*i*_(*y*) (Eq. 3).

Using these definitions, the quality function *q*_*ε*_(*y*) for endpoint *ε* can be unfolded from the following set of Fredholm equations^21^ for different radiation types *i*:

4 $${\mathcal{E}}_{i} = \int {q_{\varepsilon } (y)d_{i} (y)dy} / \int {q_{\varepsilon } (y)d_{\gamma } (y)dy} .$$

The denominator in Eq. (4), which contains the γ-ray lineal energy spectrum *d*_*γ*_(*y*), is the same for each radiation type *i*. Because we are interested only in the *shape* of the *q*_*ε*_(*y*) function, we can simplify these equations as

5 $${\mathcal{E}}_{i} = k\int {q_{\varepsilon } (y)d_{i} (y)dy,}$$

where *k* is a constant, set to 1 for simplicity.

In this study the endpoint, *ε*, is intestinal tumors in tumor-prone adenomatous-polyposis-coli APC^(1638N/+)^ mice, and the data are tumor yields (number of tumors per mouse) after exposure to space-relevant doses of energetic ^1^H, ^4^He, ^12^C, ^16^O, ^28^Si, or ^56^Fe ions^40^. These data, kindly provided by our collaborators at Georgetown University, are described below. Since this work is focused on densely-ionizing radiations, our dose–response formalism^7^ used to estimate the low-dose effect metrics, ${\mathcal{E}}_{i}$, separately includes both targeted effects (TE) due to direct traversals of cells by ionizing tracks, and non-targeted effects (NTE) caused by release of signals from directly-hit cells^6,41,42^. The other inputs needed for unfolding Eq. (5) are single-event lineal energy distributions, *d*_*i*_(*y*), for each relevant ion-energy combination, as described below.

## Methods

Our goal was to unfold an estimate of the function *q*_*ε*_(*y*) from Eq. (5), from a data set ${\mathcal{E}}_{i}$ describing the endpoint, *ε*, which represents mouse tumors induced by radiation types and doses relevant for space exploration. These data are the results of exposure to a series of different ionizing radiations, *i*, each of which we characterized by their microdosimetric energy deposition spectra *d*_*i*_(*y*). Thus, the following sections describe the generation of the biological data set ${\mathcal{E}}_{i}$, then the generation of the microdosimetric energy deposition distributions, *d*_*i*_(*y*)*,* and finally the unfolding of *q*_*ε*_(*y*) from Eq. (5).

### Mouse tumor data set

In earlier work^40^, we analyzed data on intestinal tumors in male APC^(1638N/+)^ tumor-prone mice exposed to γ rays, protons, ^4^He, ^12^C, ^16^O, ^28^Si, or ^56^Fe ions at the NASA Space Radiation Laboratory (NSRL) facility. The linear energy transfer (LET) range covered by these radiations, 0.22 to 148 keV/µm, is broad and encompasses sparsely ionizing and densely ionizing radiations. The lowest exposure of heavy ions was 5 cGy, which is in the relevant dose range for long-duration space missions^3^. The doses, LET values, and numbers of mice for each ion were as follows: unirradiated controls (68 mice), protons (1000 MeV/n; 50–120 cGy; 0.22 keV/µm, 40 mice), ^4^He (250 MeV/n; 5–50 cGy; 1.6 keV/µm, 92 mice), ^12^C (290 MeV/n; 10–200 cGy; 13 keV/µm, 60 mice), ^16^O (325 MeV/n; 5–50 cGy; 22 keV/µm, 66 mice), ^28^Si (300 MeV/n; 5–140 cGy; 69 keV/µm, 136 mice), ^56^Fe (1000 MeV/n; 5–160 cGy; 148 keV/µm, 90 mice), γ rays (5–200 cGy, 127 mice). Details of the experimental methods are described in earlier publications^43,44^.

### Generation of low dose biological response parameters for the different radiation types

We previously developed a radiation dose–response model^7^ that explicitly describes both the TE and NTE components. A summary of the model equations and fitting methods is provided here and in Appendix 1 (see Supplementary Data online).

The TE component in the APC^(1638N/+)^ mouse system is reasonably described by a linear dependence over the dose range of interest for space missions^7^. By contrast, the NTE component tends to be non-linear with a concave shape, particularly for heavy ions^7^. Dose rate effects were estimated to be minor for space flight-relevant doses of heavy ions^45^, so they are not explicitly considered here.

In our dose–response formalism^7^, the TE and NTE radiation effects at a dose *D*_*i*_ of radiation type *i* are combined in the function *M*_*ɛ*_(*D*_*i*_), which represents the total radiation response in terms of tumor yield in the mice. The function is shown in Eq. (6), where the 3 terms respectively represent the background tumor yield, the TE response, and the NTE response.

6 $$M_{\varepsilon } (D_{i} ) = B + T_{i} D_{i} + N_{i} (1 - \exp [ - s_{i} D_{i} ]).$$

Here *B* is the background parameter, *T*_*i*_ and *N*_*i*_ are the TE and NTE parameters, respectively, and *s*_*i*_ is the NTE “slope” parameter^7^. The parameter *T*_*i*_ is the slope of the linear targeted-effect dose response component. The parameter *N*_*i*_ is the “plateau” at which the NTE dose response contribution saturates, i.e., when all susceptible cells in the affected organ respond to NTE signals released by irradiated cells. The parameter *s*_*i*_ is interpreted as an exponential slope or “saturation rate” for the NTE component of the dose response.

Details of the analysis of the experimental data to generate the TE and NTE parameters for each radiation type *i* are described in Appendix 1 (see Supplementary Data online) and in our previous publication^7^. The best-fit parameter values and corresponding confidence intervals are shown in Table 1.

**Table 1:** Best-fit parameters for TE and NTE from the dose–response model (Eq. A2) fitted to mouse tumorigenesis data. Details of the method are described in the main text and in Appendix 1 (see Supplementary Data online). Notably, the *T* and *N* parameters have different units. *N* represents the “plateau” to which NTE saturate at high doses, and is therefore unitless. In contrast, *T* is a linear dose response slope for TE, with units of Gy^−1^.

Radiation type; LET (keV/µm)	TE parameter (*T*) (Gy^−1^)			NTE parameter (*N*)		
		95% CIs			95% CIs	
γ	2.77	2.47	3.04	0.62	0.33	0.87
H; 0.22	2.77	2.47	3.04	0.89	0.58	1.18
He; 1.6	2.77	2.47	3.04	1.34	1.08	1.62
C; 13	2.77	2.47	3.04	2.87	2.56	3.20
O; 22	2.77	2.47	3.04	2.68	2.34	2.94
Si; 69	8.52	8.20	8.85	3.58	3.28	3.90
Fe; 148	4.74	4.42	5.07	3.34	3.05	3.63

### Simulation of lineal energy spectra,* d*_*i*_(*y*)

We simulated single-event lineal energy (*y*) spectra for six space-relevant radiations (1000 MeV/n protons, 250 MeV/n He ions, 290 MeV/n C ions, 325 MeV/n O ions, 300 MeV/n Si ions, and 1000 MeV/n Fe ions). These ion types and energies were chosen to match those used to induce intestinal tumors in APC^(1638N/+)^ mice, as described above.

For each ion type and energy, Geant4 Monte Carlo transport software^46,47^ was used to simulate the transport of the mono-energetic beams through a Digimouse^48^ voxel mouse phantom, and each radiation track was simulated individually. During the Monte Carlo simulations, ion fluences were tallied separately for Z = 1 to Z = 28 over a fine energy grid (10,000 bins). Simulations were performed on a computing cluster using 1000 cores with adequate histories to reduce statistical uncertainties to negligible levels.

Details of the simulations are described in Appendix 2 (see Supplementary Data online). Briefly, fluences were recorded in gastrointestinal organs (bladder, stomach, spleen, pancreas, liver, kidneys) of the Digimouse. These organs were selected to match the locations where the intestinal tumors were measured in APC^(1638N/+)^ mice. The resulting particle energy spectra were used as input to the RITRACKS software^49–52^ to simulate the irradiation of a cell nucleus that is part of the GI tissues, effectively considering the primary beam and secondary particles created during the transport of the beam within tissues. Microscopic energy deposition processes were scored within spherical targets with different diameters (2, 4, 8 or 16 µm). After running multiple simulations, the full spectrum of single-event microdosimetric energy depositions was obtained. These spectra include the results of tracks that passed through the target, as well as energy depositions from tracks that missed the target and passed through nearby volumes. To check the shapes of the calculated spectra, we compared them to the predictions of a simplified triangular distribution^53^ approximation for each ion.

### Unfolding radiation quality functions, *q*_*ε*_(*y*), for TE and NTE dose–response components

As described above, in the mouse tumors system studied here the TE dose response component is reasonably approximated by a linear function (Eq. 6). The TE dose response slope for a given ion type (best-fit *T* parameter), divided by the corresponding slope for γ rays, represents the relative biological effect metric for TE (${\mathcal{E}}^{TE}$), which we used here for the ${q_{\varepsilon }}^{TE} (y)$ function estimation. ${\mathcal{E}}^{TE}$ is effectively the low-dose relative biological effectiveness for TE.

The NTE dose response component is non-linear and tends to saturate at increasing radiation doses, approaching the *N* parameter described above (Eq. 6). The *N* parameter for each ion type, divided by the corresponding *N* parameter for γ rays, is used here as the relative biological effect metric for NTE, ${\mathcal{E}}^{NTE}$, for the ${q_{\varepsilon }}^{NTE} (y)$ function estimation.

This approach enabled us to generate two low-dose relative biological effect metrics, ${\mathcal{E}}^{TE}$ and ${\mathcal{E}}^{NTE}$, based on best-fit values for the *T* and *N* parameters in the dose response model. The radiation quality function *q*_*ε*_(*y*)_,_ which was applied to both metrics, is described by the following equation, where *y* is lineal energy and *k*_1_–*k*_3_ are adjustable parameters (positive numbers, potentially different for TE and NTE metrics):

7 $$q_{\varepsilon } (y) = \exp \left[ {k_{1} \left( \frac{y}{100} \right)^{{k_{2} }} - k_{3} \left( \frac{y}{100} \right)^{{\left[ {1 + k_{2} } \right]}} } \right].$$

The mathematical structure of this *q*_*ε*_(*y*) function was chosen to satisfy the following properties, based on available knowledge of radiation quality dependences: (1) At low lineal energies (*y* < 5 keV/µm), ${\mathcal{E}}^{TE}$ or ${\mathcal{E}}^{NTE}$ should be approximately unity; (2) As the lineal energy increases above 5 keV/µm, ${\mathcal{E}}^{TE}$ or ${\mathcal{E}}^{NTE}$ should increase smoothly; (3) At high lineal energies (*y* ≥ 50 keV/µm), ${\mathcal{E}}^{TE}$ or ${\mathcal{E}}^{NTE}$ should peak and then begin to flatten out or decrease at higher *y* values, due to energy deposition saturation. This saturation effect occurs when the energy deposition within the target is larger than that needed to cause the biological effect, so the remaining energy deposition is effectively “wasted”^54^.

Equation (7) represents a simple 3-parameter function that can satisfy these criteria, but alternative formulations for *q*_*ɛ*_(*y*) are, of course, possible. Exploratory calculations with a variety of different functional forms produced marginally worse fits to our data.

We substituted the mathematical structure of *q*_*ε*_(*y*) from Eq. (7) into Eq. (5) to generate predictions for both *ε* metrics (${{\mathcal{E}}^{TE}}_{pred}$ and ${{\mathcal{E}}^{NTE}}_{pred}$) for each radiation type, *i*. The sum of squared differences between these predictions and corresponding observations was then minimized to find best-fit values for the unknown parameters *k*_1_–*k*_3_ for each of the two metrics. The functions to be minimized (${F^{X}}_{opt}$, where *X* is either TE or NTE) are described by the following equation, where ${{\mathcal{E}}^{X}}_{obs}$ represents either ${\mathcal{E}}^{TE}$ or ${\mathcal{E}}^{NTE}$ estimates based on parameter values from our dose–response model fitted to the mouse tumor data set:

8 $${F^{X}}_{opt} = \sum\nolimits_{i} {\left[ {{{\mathcal{E}}^{X}}_{pred} - {{\mathcal{E}}}_{obs} } \right]^{2} /\left[ {{{\mathcal{E}}^{X}}_{obs\; \, CI} } \right]^{2} ,}$$

where ${\mathcal{E}}_{obs\,CI}$ is the 95% confidence interval (CI) width (upper bound value minus lower bound value) for the respective ${{\mathcal{E}}^{X}}_{obs}$ values.

We minimized ${F^{X}}_{opt}$ using a sequential quadratic programming algorithm implemented in Maple 2020 software. The parameters *k*_1_–*k*_3_ were restricted to the range between 0 and 10 to produce smooth curves without excessively sharp peaks. To find the global optimum solution, we performed the optimization procedure 300 times starting from random initial values for parameters *k*_1_–*k*_3_. The best solutions (lowest ${F^{X}}_{opt}$ values) were stored and used to generate best-fit predictions for the *q*_*ε*_(*y*) functions for TE and for NTE.

Best-fit values for parameters *k*_1_–*k*_3_ were then substituted into Eq. (5) to produce predicted ${\mathcal{E}}_{TE}$ and ${\mathcal{E}}_{NTE}$ low-dose metrics for each radiation type, based on the *d*_*i*_(*y*) spectra for each radiation type *i*. These predictions were compared with the same metrics derived directly from fitting the dose–response model to the measured data, using coefficient of determination (R^2^) and root mean squared error (RMSE) metrics.

### TE and NTE predictions using lineal energy spectra for the space environment and the Mars surface

Calculations of lineal energy distributions for the space environment and on the Mars surface were obtained from Northum et al*.*^55^. These spectra were digitized from figures in Northum et al.^55^ and fitted using polynomial functions. The normalized *d*(*y*) functions were combined with the best-fit *q*_*ε*_(*y*) functions using Eq. (5), to produce predicted ${\mathcal{E}}^{TE}$ and ${\mathcal{E}}^{NTE}$ low-dose metrics for the space environment and the Mars surface.

## Results

The best-fit radiation responses for mouse tumors induced by H, He, C, O, Si, and Fe ions, and γ rays for comparison, modeled using Eqs. (6) and (A2), are shown in Supplementary Fig. 1 (see Supplementary figures online). These dose responses were subsequently used as input for the *q*_*ɛ*_(*y*) calculations. Comparisons of these dose responses with the tumor yield data show that non-linearity occurs at low doses, in the range of 0, 0.05, and 0.1 Gy. This non-linearity is particularly prominent for the heavier ions (Si and Fe) and is attributed to NTE in our dose–response model^7^. Best-fit parameters for the model are shown in Table 1. As described in Appendix 1 (see Supplementary Data online), the *T* parameters for particle radiations (H, He, C, O, Si, and Fe ions) were restricted to be no less than the best-fit value for γ rays. The parameter *s* was assumed to be the same for all radiation types and its best-fit value was determined as 38.7 (95% CI 38.3, 39.0) Gy^−1^.

**Figure 1:**
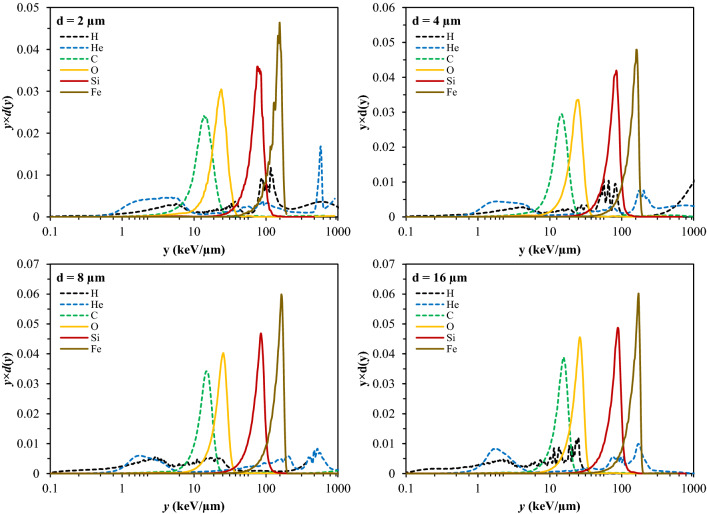
Calculated dose-normalized distributions of microdosimetric energy deposition as a function of lineal energy (*y*) for different ion types (energies given in the text) and different sized spherical target diameters (*d* = 2, 4, 8, or 16 µm). Normalized *y* × *d*(*y*) spectra are shown, such that equal areas under the *y* × *d*(*y*) curve between any two *y* values correspond to equal doses^38^. The spectra for the same ions, plotted using a linear y-axis scale, are shown in Supplementary Fig. 2 to improve visualization of different spectrum components.

The dose-normalized lineal energy spectra for each ion type and each spherical target diameter are shown in Fig. 1 and in Supplementary Fig. 2 (see Supplementary figures online). The spherical target diameter, within the investigated range of 2–16 µm, appeared to have only a moderate effect on the spectra. These spectra have a complicated structure, which is particularly visible in the logarithmic plots. However, dose-weighted mean lineal energy values from the spectra for the heavier ions (C, O, Si, Fe) were reasonably close to predictions based on the simple triangular distribution approximation^53^, where the dose-weighted mean lineal energy is approximated by (9/8) × LET, where LET is linear energy transfer. For example, taking the spectra for targets with 16 µm diameter, for C ions the triangular distribution estimated the dose-weighted mean lineal energy to be 14.6 keV/µm, whereas the simulated *d*(*y*) spectrum generated a value of 11.2 keV/µm. For O ions the respective values were 24.8 vs. 18.1 keV/µm, for Si 77.6 vs. 60.5 keV/µm, and for Fe 166.5 vs. 108.3 keV/µm. For light ions the differences were larger: for H 0.2 vs. 1.1 keV/µm, for He 1.8 vs. 3.5 keV/µm.

**Figure 2:**
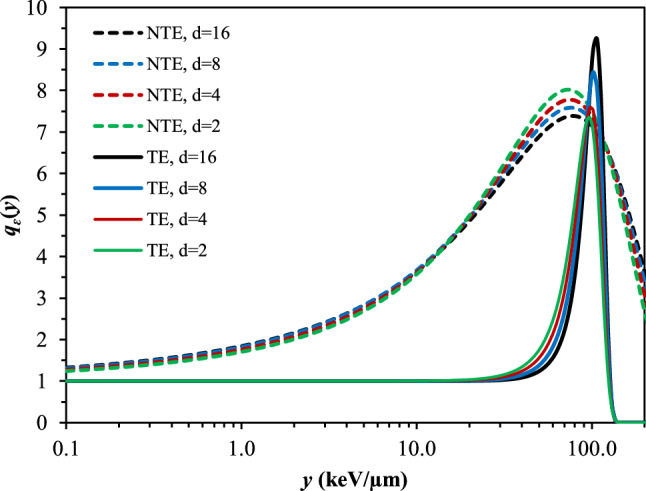
Best-fit radiation quality functions *q*_*ɛ*_(*y*) for targeted effects (TE, solid curves) and non-targeted effects (NTE, dashed curves), for different spherical target diameters (d in µm).

**Figure 3:**
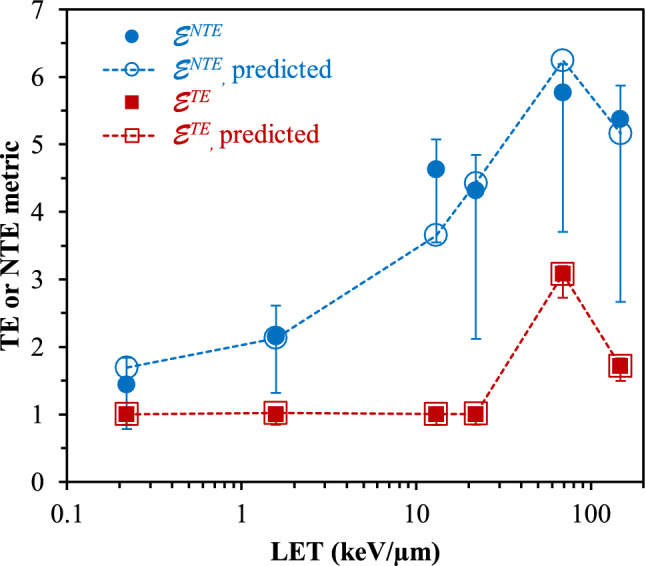
TE and NTE metrics, ${\mathcal{E}}^{TE}$ and ${\mathcal{E}}^{NTE}$ for the 6 radiation types. These metrics were derived from fitting the dose–response model to the measured data. Corresponding predictions were produced based on *d*(*y*) and best-fit *q*_*ɛ*_(*y*) functions, as explained in the text. Error bars represent 95% CIs. Predictions are shown for the spherical target diameter of 16 µm, and they were similar for the other investigated diameters. Straight lines connecting the points are shown only to guide the eye.

The central results of this study—the best-fit unfolded *q*_*ɛ*_(*y*) functions for the TE and the NTE metrics—are shown in Fig. 2. Corresponding parameters for the *q*_*ɛ*_(*y*) functions, and comparisons of the observed and the predicted low-dose relative biological effect metrics for each radiation type, are shown in Tables 2, 3 and in Fig. 3.

**Table 2:** Best-fit parameter values for low doses from the radiation quality functions of lineal energy *q*_*ɛ*_(*y*) for the ${\mathcal{E}}^{TE}$ and ${\mathcal{E}}^{NTE}$ metrics, for each spherical target diameter. Agreement between observed and best-fit predicted relative biological effect metrics over all radiation types was assessed by R^2^ and RMSE.

Biological effect metric	Target diameter (µm)	Best-fit quality function parameters			Comparison of observed and predicted biological effect metrics	
		*k*_1_	*k*_2_	*k*_3_	R^2^	RMSE
${\mathcal{E}}^{TE}$	16	10.00	4.84	7.85	1.000	0.009
	8	10.00	4.24	7.88	1.000	0.010
	4	10.00	3.77	7.97	1.000	0.017
	2	10.00	3.42	8.03	1.000	0.027
${\mathcal{E}}^{NTE}$	16	2.91	0.34	0.95	0.919	0.465
	8	3.01	0.35	1.02	0.913	0.483
	4	3.13	0.37	1.13	0.895	0.531
	2	3.28	0.39	1.26	0.879	0.574

**Table 3:** Low dose TE and NTE metrics, ${\mathcal{E}}^{TE}$ and ${\mathcal{E}}^{NTE}$ for the six radiation types. Comparison of these metrics derived from fitting the dose–response model to the measured data (Table 1) vs. corresponding predictions based on *d*(*y*) and best-fit *q*_*ɛ*_(*y*) functions (Table 2). Predictions are shown for each spherical target diameter (d, in µm). Details of the methods are described in the text.

Ion, LET (keV/μm)	${\mathcal{E}}^{TE}$ from dose–response model			${\mathcal{E}}^{NTE}$ from dose–response model			Predicted ${\mathcal{E}}^{TE}$ based on TE *q*_*ɛ*_(*y*) function				Predicted ${\mathcal{E}}^{NTE}$ based on NTE *q*_*ɛ*_(*y*) function			
		95% CIs			95% CIs		d = 16	d = 8	d = 4	d = 2	d = 16	d = 8	d = 4	d = 2
H, 0.22	1.00	0.85	1.10	1.44	0.60	1.90	1.00	1.00	1.03	1.04	1.69	1.72	1.82	1.86
He, 16	1.00	0.85	1.10	2.15	1.08	2.60	1.02	1.02	1.01	1.02	2.13	2.12	2.08	2.07
C, 13	1.00	0.85	1.10	4.63	2.43	5.16	1.00	1.01	1.01	1.01	3.65	3.62	3.52	3.45
O, 22	1.00	0.85	1.10	4.31	2.25	4.73	1.01	1.02	1.03	1.04	4.43	4.39	4.29	4.23
Si, 69	3.08	2.73	3.20	5.77	3.06	6.27	3.08	3.08	3.07	3.06	6.24	6.27	6.27	6.31
Fe, 148	1.71	1.50	1.83	5.37	2.85	5.85	1.71	1.71	1.71	1.71	5.16	5.15	5.13	5.06

The *q*_*ɛ*_(*y*) functions for the ${\mathcal{E}}^{TE}$ and ${\mathcal{E}}^{NTE}$ metrics (Fig. 2) both peak at lineal energies between 50 and 100 keV/µm, but their overall shapes are clearly different, particularly at intermediate lineal energy values (~ 5 to 50 keV/μm). This difference in shape was essentially determined by the sharp difference in ${\mathcal{E}}^{TE}$ values for Si vs. Fe ions (Table 3), while by contrast the ${\mathcal{E}}^{NTE}$ values changed more gradually between radiation types (Table 3).

The target diameter had only a small effect on the *q*_*ɛ*_(*y*) functions (Fig. 2), however, somewhat better fit statistics (higher R^2^ and lower RMSE, Table 2) were obtained for the largest diameter of 16 µm. A visual comparison of the observed and the predicted relative biological effect values for TE and NTE is shown in Fig. 3, based on the best-fit *q*_*ɛ*_(*y*) functions for targets with diameter of 16 µm.

As an example of potential utility of this approach, we applied the best-fit *q*_*ɛ*_(*y*) functions for TE and NTE to calculated^55^ microdosimetric lineal energy deposition distributions for the space environment and for the surface of Mars. The resulting predicted ${\mathcal{E}}^{TE}$ and ${\mathcal{E}}^{NTE}$ low-dose metrics for the space environment and for the Mars surface are shown in Table 4. The metrics were somewhat higher for the space environment than for the Mars surface because the space environment spectrum contained a larger contribution of high lineal energies^55^. Of interest is that the low-dose metrics for NTE were 2–3 times higher than those for direct TE.

**Table 4:** Predicted ${\mathcal{E}}^{TE}$ and ${\mathcal{E}}^{NTE}$ metrics for the space environment and for the Mars surface: predictions are based on published calculated lineal energy distributions for the space environment and for the Mars surface (from Northum et al.^55^), combined with our best-fit *q*_*ɛ*_(*y*) functions for TE and NTE. Details of the methods are described in the text.

Location	Target diameter (µm)	Predicted ${\mathcal{E}}^{TE}$	Predicted ${\mathcal{E}}^{NTE}$
Free space	16	1.20	3.46
	8	1.20	3.48
	4	1.21	3.44
	2	1.22	3.41
Mars surface	16	1.07	2.69
	8	1.08	2.70
	4	1.08	2.65
	2	1.09	2.61

## Discussion

Currently, two approaches can be used to estimate radiation quality factors, and thus biological effective doses, for a complex low/intermediate dose radiation environment of space, where total expected doses are < 1 Gy. One is a fluence-based risk cross-section approach, which requires a very detailed in silico characterization of the radiation field, and thus, for example, cannot realistically be used for in situ quality factor estimates. By contrast, the microdosimetric approach, which uses distributions of microdosimetric energy deposition that are weighted with an empirically determined biological weighting function, is conceptually and practically simpler.

To apply the microdosimetric approach, as exemplified by Eqs. (1, 2), to the space environment, two quantities are required: first the radiation environment must be characterized through the microscopic energy deposition distribution, *d*(*y*) and, secondly, a consensus biological weighting factor, *Q*(*y*), must be available.

In practice, portable rugged microdosimeters can be used in a space flight environment to continuously measure *d*(*y*)*,* and several such devices have already been built and tested^17–20^. In addition, a ground-based calculation of a time-averaged *d*(*y*) function for the relevant space environment, leading to a predicted time-averaged quality factor, will be useful for mission planning purposes.

The second requirement is the consensus biological weighting function, *Q*(*y*)*,* which could be applied to the microdosimeter software (see Eqs. 1, 2) to produce a continuous in situ estimation of the quality factor and thus the biologically effective dose (dose equivalent or equivalent dose). It is important to note that, as with all quality factors and radiation weighting factors^8–11^, the *Q*(*y*) biological weighting function should be a consensus weighted average of a number of functions, *q*_*ε*_(*y*)*,* each of which are determined for specific relevant biological endpoints, *ε*. Currently there is no such consensus biological weighting function, Q(y) and this paper can be seen as providing a “roadmap” towards the generation of such a function through estimation of a series of relevant *q*_*ε*_(*y*) functions.

To demonstrate the feasibility of this approach, we estimated a biological weighting function, *q*_*ε*_(*y*), for one specific relevant endpoint, intestinal tumor yields in APC^(1638N/+)^ mice, which we unfolded from experimental results after a variety of heavy-ion exposures, together with corresponding calculated *d*_*i*_(*y*) spectra for the heavy ions.

In our analysis, separate biological weighting functions, *q*_*ɛ*_(*y*), were estimated for TE and NTE, and the resulting functions were substantially different in shape (Fig. 2). Specifically, the NTE *q*_*ɛ*_(*y*) function starts to rise above its values at low *y* (i.e. the value for γ rays) by around 1 keV/µm, whereas the TE *q*_*ɛ*_(*y*) function started to rise substantially only at lineal energy values ≥ 50 keV/µm. These differences suggest that the intermediate *y* region between approximately 1 and 50 keV/µm is clearly more effective in generating NTE compared to TE.

These biological weighting functions, *q*_*ɛ*_(*y*), for TE and NTE were folded with microdosimetric energy-deposition spectra that were estimated for the space environment and for the Mars surface, producing estimates of the low-dose relative biological effect metrics at both locations. Of interest is that the predicted low-dose metrics were two- to three-fold larger for NTE than for TE. This suggests that NTE may play an important role in the response to densely-ionizing radiations such as in space, and should be considered explicitly in quantifying the radiation related health effects from space missions.

It is emphasized again that the quality function *q*_*ɛ*_(*y*) estimated in this current work refers to a single specific endpoint (yields of intestinal tumors in APC^(1638N/+)^ mice), and *q*_*ɛ*_(*y*) functions for other relevant endpoints must be estimated to produce the data set needed for a consensus *Q*(*y*) evaluation. *q*_*ɛ*_(*y*) spectra have already been estimated for several endpoints^21,56,57^, and several other “relevant” data sets are available that could be analyzed to produce *q*_*ɛ*_(*y*) functions, both for cancer^4,58^ and non-cancer endpoints^59–62^. Although *q*_*ɛ*_(*y*) functions can be unfolded from the results of a series of biological studies of different LET, as was done here, *q*_*ɛ*_(*y*) functions can also be unfolded from a series of radiobiological studies using a single high-LET radiation at different doses^57^.

We conclude that the microdosimetric approach to estimate quality factors is indeed practical to use for in situ space flight environments and, in conjunction with continuous measurements of microdosimetric spectra, can be used to produce a continuous assessment of quality factors and biological effective doses such as the dose equivalent.

## Supplementary Information

Supplementary Information.

Supplementary Figures.
