# Supplementary material for: A practical approach for continuous in situ characterization of radiation quality factors in space
Source: Sci Rep. 2022 Jan 27;12:1453. doi: 10.1038/s41598-022-04937-1 (PMC8795169; doi:10.1038/s41598-022-04937-1)
Supplement: Supplementary file 1 — Supplementary Information. [file 41598_2022_4937_MOESM1_ESM.docx]

**A Practical Approach for Continuous *In Situ* Characterization of
Radiation Quality Factors in Space**

Igor Shuryak^1*^, Tony C. Slaba^2^, Ianik Plante^3^, Floriane Poignant^4^, Steven R. Blattnig^2^, David J. Brenner^1^

1. Center for Radiological Research, Columbia University Irving Medical Center, New York, NY10032

2. NASA Langley Research Center, Hampton, VA 23681

3. KBR, Houston, TX 77058

4. National Institute of Aerospace, Hampton, VA 23666

^*^ Corresponding author: Igor Shuryak, M.D., Ph.D.

Center for Radiological Research, Columbia University,

630 West 168^th^ St., New York, NY 10032

Phone: 212-305-2405; Fax: 212-305-3229

E-mail: [is144@cumc.columbia.edu](mailto:is144@cumc.columbia.edu)

**APPENDICES**

**Appendix 1: Dose Response Model and Model Fitting**

***1A: Dose Response Model***

In our formalism, the TE and NTE radiation effects at a dose *D* of radiation type *i* are combined in the function *M_ɛ_(D_i_)*, which represents the total radiation response in terms of the number of intestinal tumors per mouse. The function is provided in Eq. (6) in the main text. It contains the background tumor yield parameter (*B*), the TE and NTE parameters (*T_i_* and *N_i_*, respectively), and the NTE “slope” parameter (*s_i_*).

***1B: Tumor Number Variability***

The APC^(1638N/+)^ mouse data ^1,2^ suggest that the number of tumors per mouse does not follow Poisson distribution. Both “overdispersion” and “underdispersion”, compared with the Poisson distribution, are encountered in this data set, depending on radiation type and dose. For example, after 0.05 Gy of γ rays, the mean number of tumors per mouse (µ) was 3.84 and variance mean (V/µ) was 0.57, indicating notable underdispersion relative to the Poisson distribution, where V/µ = 1 by definition. By contrast, after 1.4 Gy of Si ions the mean number of tumors per mouse was 23.28 and V/µ was 4.58, indicating notable overdispersion.

To describe this complexity in the variability of the number of tumors, we used the following customized weighted negative binomial (WNB) distribution, where *k* is the number of tumors per mouse, *P_WNB_*(*k*) is the probability of *k*, *M_ɛ_* is the radiation response function (from Eq. 6 in the main text), *r* and *q* are parameters that describe the variance, Γ is the gamma function, and *Y* = *k* + 1/*r*:

$P_{WNB}\left( k \right)=\frac{{[\left( 1+r M_{ɛ} \right)}^{-Y} r^{k} M_{ɛ}^{\left( k-1 \right)} \Gamma\left( Y \right) \left( M_{ɛ}+k q \right)]}{\left[ \Gamma\left( 1+k \right) \Gamma\left( \frac{1}{r} \right) \left( 1+q \right) \right]}$ (A1)

The mean number of tumors per mouse (µ), based on Eq. (A1), is as follows:

$\mu=\left[ M_{ɛ}+q \left( 1+M_{ɛ} \left( 1+r \right) \right) \right]/(1+q)$ (A2)

The variance/mean ratio for the WNB distribution has the following solution:

$\frac{V}{\mu}=\left( 1+r M_{ɛ} \right) \frac{\left[ q+M_{ɛ} \left( 1+q^{2} \left( 1+r \right)+2 q \left( 1+r \right) \right) \right]}{[\left( 1+q \right) \left( q+M_{ɛ} \left( 1+q \left( 1+r \right) \right) \right)]}$. (A3)

***1C: Data Fitting***

The dose response model parameters (*T*, *N*, *s,* and *r*) were estimated by fitting the model to the data using maximum likelihood techniques, as described below. Importantly, we sought to reduce the number of freely adjustable parameters to simplify the model structure and the interpretation of model predictions. For this reason, we did not allow the remaining parameters *B* and *q* to be freely adjustable, but instead estimated both parameters based on observed data for unirradiated control mice. Based on these assumptions, the following equation describes the relationship of these parameters with the mean background tumor yield (µ*_bac_*, observed value = 3.279).

$\mu_{bac}=\left[ B+q (1+B (1+r)) \right]/(1+q)$. (A4)

This equation (Eq. A4) can be solved for *B* as function of µ*_bac_*, as follows:

$B_{sol}=\left[ \left( \mu_{bac}-1 \right) q+\mu_{bac} \right]/\left[ 1+q (1+r) \right]$. (A5)

The relationship between the parameters of interest and the background variance in tumor counts per mouse (*V_bac_*, observed value = 2.63) is described as follows, where *B_sol_* is based on Eq. (A5):

$V_{bac}=\frac{\left[ \left( \left( 1+q^{2} \left( 1+r \right)+2 q \left( 1+r \right) \right) B_{sol}+q \right) \left( 1+r B_{sol} \right) \right]}{{(1+q)}^{2}}$ (A6)

There are two solutions for parameter *q* based on Eq. (A6). We discarded the negative solution. The retained positive solution has a singularity in the positive *r* range, so we approximated it with the following smooth function (*q_sol_A_*) of *r*:

$q_{sol_{A}}=500 \left( 1-\exp\left[ -\frac{\exp\left[ a_{1}+a_{2} r-a_{3} r^{2}+a_{4} r^{3} \right]}{500} \right] \right),$ (A7)

where *a*_1_ = 1.34, *a*_2_ = 26.85, *a*_3_ = 20.95, *a*_4_ = 1843.1.

We substituted *q_sol_A_* from Eq. (A7) into Eq. (A5) to obtain the following approximate explicit solution for *B* (*B_sol_A_*) as function of *r*:

$B_{sol\_A}=\left[ \left( \mu_{bac}-1 \right) q_{sol\_A}+\mu_{bac} \right]/\left[ 1+q_{sol\_A} (1+r) \right]$ (A8)

We generated these solutions for parameters *B* and *q* (*B_sol_A_* and *q_sol_A_*) from Eqs. (A7 – A8) for each radiation type, based on the best-fit value of parameter *r*. In this manner, the model was substantially simplified by allowing the fitted value for one parameter (*r*) to determine the values of two other parameters (*B* and *q*).

We fitted the model to individual tumor numbers for each mouse by maximizing the log likelihood function (*LL*) over all the data for all the studied radiation types combined (controls, γ rays, H, He, C, O, Si, and Fe ions), using the sequential quadratic programming algorithm implemented in Maple 2020 software. To improve biological realism, we implemented the following constraints: First, all parameters were restricted to non-negative values and, second, the *T* parameters for particle radiations (H, He, C, O, Si, and Fe ions) were restricted to be no less than the best-fit value for γ rays. This restriction was mainly relevant for those heavy ions (*e.g.* He, O) for which doses >1 Gy were not available, because, for the radiations where these doses were available, such high doses influenced the *T* parameter. To maximize the probability of finding a global (rather than a local) optimum for the model fit, the model was refitted 2000 times using randomly chosen initial parameter values, and the best-fit solution among all these attempts was selected as the optimum.

To simplify the model further, we determined whether common parameters could be used to assess all radiation types by comparing the performances of model variants with a selected common parameter *vs*. different parameter for each radiation type. We performed the comparisons using the Akaike information criterion with sample size correction (AICc)^3,4^, and found that a common *s* parameter was sufficient for all radiation types, common *T* and *r* parameters were sufficient for γ, H, He, C and O, and different *T* and *r* parameters were required for Si and Fe, whereas parameter *N* differed for each radiation type. This model variant was used for the analyses described below.

***1D: Uncertainty Estimation for Model Parameters***

Uncertainties (95% confidence intervals, CIs) were estimated for each adjustable model parameter using the following Monte Carlo procedure: Multiple (>7,500) randomly-selected combinations of parameter values producing model fits that fell within the 95% CIs of the best fit (assessed by profile likelihood) were generated and stored. These parameter combinations were then used to generate distributions of values for each parameter. The minimum and maximum values of each distribution generated by Monte Carlo were used as estimates for the 95% CIs of the selected parameter.

**Appendix 2: Simulations of Microdosimetric Energy Deposition Spectra for Each Ion**

We entered the simulated ion fluence $\phi\left( Z,E \right)$ into the stochastic radiation track structure code RITRACKS^5^ to simulate target irradiation. For these simulations, spherical targets were encompassed into parallelepiped irradiation volumes. The number of tracks of each ion type was calculated by integrating the spectra modulated by the LET, *i.e*.

$$\begin{aligned} \phi(Z)=\int_{E} \phi\left( Z,E \right) dE.\#\left( A9 \right) \end{aligned}$$

Because the values $\phi\left( Z,E \right)$ are tabulated at certain energies, this integral was evaluated numerically. Furthermore, the contribution of each ion type to the dose was calculated as

$$\begin{aligned} D(Z)=1.6 {10}^{9}\int_{E} \phi\left( Z,E \right) LET(Z,E)dE.\#\left( A10 \right) \end{aligned}$$

The dose was calculated by summing the contributions for each ion, *i.e*. $D_{tot}=\sum_{Z} D(Z)$. To simulate a given dose $D_{req}$, the fluences were multiplied by the ratio ${D_{req}/D}_{tot}$. The number of tracks for each Z was obtained by sampling a Poisson distribution using $\lambda=({D_{req}/D}_{tot})\phi(Z)A$, where A is the area of the irradiated volume. For each track, the energy was determined using a rejection method^6^. Essentially, a random energy $E_{rnd}$ was generated between $E_{min}$ and $E_{max}$, which are the minimum and maximum energies in which the spectra are defined. A random number U was drawn between 0 and the maximum value of the spectra for Z, $\phi_{max}\left( Z,E \right)$. If $U\leq\phi(Z,E_{rnd})$, the energy value $E_{rnd}$ was accepted. The process was repeated until an energy was accepted.

To account for the energy that is deposited outside of the irradiated volume by delta rays, and the energy deposition from tracks generated outside the irradiated volume, periodic boundary conditions (PBCs) were used. Briefly, an electron that leaves the irradiated volume was returned to the opposite side of the volume with the identical direction vector. Without PBCs, the dose to the irradiated volume and the target were significantly lower than expected, as energy is lost by energetic delta-electrons leaving the volume that is not compensated by entering delta-electrons generated by tracks crossing neighbor volumes, as expected for a volume that is part of a tissue at charge equilibrium. With PBCs, the dose to the target is in good agreement with the expected dose, as it effectively mimic the contribution of delta-electrons coming from tracks traversing neighbor volumes.

**References**

1. Kumar, S., Suman, S., Fornace, A. J. & Datta, K. Space radiation triggers persistent stress response, increases senescent signaling, and decreases cell migration in mouse intestine. *Proc. Natl. Acad. Sci.* **115**, E9832–E9841 (2018).

2. Suman, S. *et al.* Relative biological effectiveness of energetic heavy ions for intestinal tumorigenesis shows male preponderance and radiation type and energy dependence in APC1638N/+ mice. *Int. J. Radiat. Oncol.* **95**, 131–138 (2016).

3. Wagenmakers, E.-J. & Farrell, S. AIC model selection using Akaike weights. *Psychon. Bull. Rev.* **11**, 192–196 (2004).

4. Burnham, K. P. & Anderson, D. R. P values are only an index to evidence: 20th- vs. 21st-century statistical science. *Ecology* **95**, 627–30 (2014).

5. Plante, I. & Cucinotta, F. Monte-Carlo simulation of ionizing radiation tracks. in *Application of Monte Carlo methods in biology, medicine and other fields of science* 315–356 (InTech, 2011).

6. von Neumann, J. Various techniques used in connection with random digits. in *Monte Carlo methods* 36–38 (National Bureau of Standards, 1951).
