# Supplementary material for: A practical approach for continuous in situ characterization of radiation quality factors in space
Source: Sci Rep. 2022 Jan 27;12:1453. doi: 10.1038/s41598-022-04937-1 (PMC8795169; doi:10.1038/s41598-022-04937-1)
Supplement: Supplementary file 2 — Supplementary Figures. [file 41598_2022_4937_MOESM2_ESM.docx]

**A Practical Approach for Continuous *In Situ* Characterization of
Radiation Quality Factors in Space**

Igor Shuryak^1*^, Tony C. Slaba^2^, Ianik Plante^3^, Floriane Poignant^4^, Steven R. Blattnig^2^, David J. Brenner^1^

1. Center for Radiological Research, Columbia University Irving Medical Center, New York, NY10032

2. NASA Langley Research Center, Hampton, VA 23681

3. KBR, Houston, TX 77058

4. National Institute of Aerospace, Hampton, VA 23666

^*^ Corresponding author: Igor Shuryak, M.D., Ph.D.

Center for Radiological Research, Columbia University,

630 West 168^th^ St., New York, NY 10032

Phone: 212-305-2405; Fax: 212-305-3229

E-mail: [is144@cumc.columbia.edu](mailto:is144@cumc.columbia.edu)

**Supplementary Figure 1.** **Comparison of model fits (Eq. A2 in Supplementary Data online, red curves) with the data (black points, blue circles) for mouse tumorigenesis induced by H, He, C, O, Si, or Fe ions, or γ rays.** Black points represent the number of tumors in individual mice. Blue circles and bars represent mean values and standard errors, and red curves represent model fits. Two of the Si irradiated mice had >40 tumors (50 and 53), but these points are not shown in the Si panel below to improve visualization at lower y-axis values.

**Supplementary Figure 1 (continued)**

**Supplementary Figure 2.** **Calculated dose-normalized microdosimetric energy deposition distributions *d(y)* as function of lineal energy (*y*) for different ion types (energies given in the text) and spherical target diameters (*d* = 2, 4, 8, or 16 µm).**
